# A Versatile Tool for Stable Inhibition of microRNA Activity

**DOI:** 10.3390/biology2030861

**Published:** 2013-06-28

**Authors:** Paride Pelucchi, Valeria Tria, Valentina Martino, Davood Sabour, Giovanni Bertalot, Stefano Molgora, Mira Palizban, Martin Götte, Ileana Zucchi, Rolland A. Reinbold

**Affiliations:** 1 Institute of Biomedical Technologies, National Research Council, 20090 Segrate-Milan, Italy; E-Mails: paride.pelucchi@itb.cnr.it (P.P.); valeria.tria@itb.cnr.it (V.T.); valentina.martino@itb.cnr.it (V.M.); stefano.molgora@itb.cnr.it (S.M.); mira.palizban@uni-muenster.de (M.P.); 2 Center of Physiology and Pathophysiology, Institute of Neurophysiology, University of Cologne, Robert-Kock Str. 39, D-50931, Cologne, Germany; E-Mail: dsabour@uni-koeln.de; 3 Pathology and Anatomy Unit, Manerbio, Desenzano d/G, 25025 Manerbio, Italy; E-Mail: g.bertalot@libero.it; 4 University of Münster Medical Center, Gynecology and Obstetrics, Münster 48149, Germany; E-Mail: martingotte@uni-muenster.de

**Keywords:** miRNA, lentivirus, miR-inhibitor

## Abstract

MicroRNAs (miRNAs) are a class of small RNAs (18–22 nt) that post transcriptionally regulate gene expression by binding to complementary sequences on target mRNAs, resulting in translational repression or target degradation and gene silencing. As aberrant expression of miRNAs is implicated in important diseases including cancer miRNA-based therapies are under intensive investigation. We optimized strategies to stably or conditionally generate miRNA inhibitors for a continuous block of miRNA activity that allows for probing miRNA function in long-term cell culture experiments, cancer xenografts, 3D tissue models and for *in vivo* studies with transgenic organisms.

## 1. Introduction

MicroRNAs (miRNAs, miRs) are a class of evolutionary conserved small (18–22 nt) non-coding RNAs. MiRs, discovered in 1993 in *C. elegans*, play a pivotal role in biological processes in normal development and disease in both plants and animals [1,2,3,4]. MiRs accomplish their functions by guiding the interaction of the RNA-induced silencing complex (RISC) to complementary or partially complementary sequences in target mRNAs, thereby regulating gene expression through translation inhibition or degradation of transcripts [5,6]. A miR can regulate multiple transcripts depending on the cell type, function and differentiation stage, and a single transcript can be regulated by more than one miR [7,8,9]. Inference of microRNA function usually cannot be derived exclusively through the prediction of their candidate targets. Even with the compilation of predicted targets and pathways the targets regulate, the functions of miRs need to be determined experimentally [10]. To identify function, expression levels or activity of miRs are modulated to observe the effects on cells. A preferred approach is to inhibit miR activity rather than up-regulate miR expression, since the former may reveal the function of the miR at physiological levels in cells. As miRNA activity is dependent on the relative concentration of the miR and its targets, up regulating a miR at levels higher than physiological may result in repression of target mRNAs that normally would not be targeted in normal conditions [11].

Various approaches are utilized to inhibit microRNA activity in cells, the majority of which rely on short-term inhibition of miR activity based on the effects of transient delivery into cells of oligos with miR-blocking activity. Fewer strategies have been developed in which stable expression of sequences complementary to miRs act as microRNA inhibitors, designated as decoys, miR target (miRT) sequences, erasers and antagomirs [12,13,14,15,16]. Whether transient and stable, inhibition of miRNA activity is based on oligos that contain nucleotide sequences that are either perfectly antisense to or contain mismatches in the middle nucleotide-positions of microRNA binding sites. Use of mismatches in oligo design is preferable as this prevents Ago2-mediated endonucleolytic cleavage of the oligo itself [17,18,19].

Here we describe a robust method that we developed to generate highly efficient stable expressed miR-blocking transcripts in cells, that we call “miR-scavengers”. This method was designed to achieve sustainable microRNA blocking activity enabling the analysis of the effects of miR loss-of function in long-term studies both *in vitro* and *in vivo*. Our approach can also be adjusted for conditionally inducing microRNA blocking activity utilizing specific cell type or tetracycline-inducible promoters for miR-scavenger expression.

## 2. Results and Discussion

**Figure 1:**
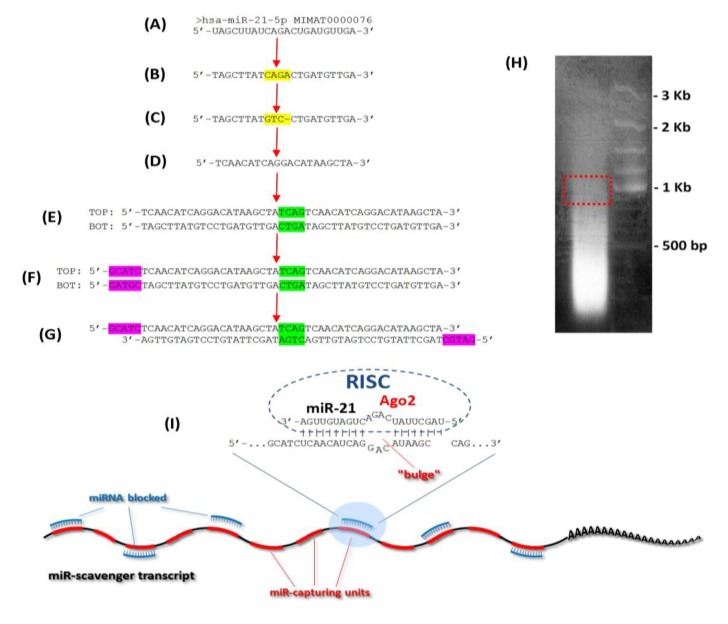
Schematic representation of the miR-scavenger protocol, illustrated here with hsa-miR-21. (**A**) Sequence of the mature miR-21 retrieved from the miRBase database [27]. (**B**) DNA sequence of miR-21 (U is substituted with T). (**C**) Four nucleotides (CAGA, in yellow) in the central portion of the sequence are changed to form a “bulge-like” structure. (**D**) Inverse complementary sequence that binds to the mature miR-21 (the “miR-capture sequence unit”). (**E**) Duplication of the miR-capture sequence unit to form a tandem sequence with a spacer (green) between two units. TOP and BOT are complementary strands allowing the formation of the double-stranded oligo. (**F**) Addition of the sticky ends sequences (purple) at the 5' of both oligos to produce the miR-scavenger. (**G**) The annealed double stranded oligos ready to be concatemerized. (**H**) Agarose gel electrophoresis of the concatemerized product: red box indicates the portion excised and purified to be used for the cloning procedure. (**I**) Mechanism of action of each miR-capture-unit and structure of a “miR-scavenger transcript”. Each microRNA molecule (blue) binds a “miR-capturing-unit” (red) by the RNA-induced silencing complex (RISC). A “bulge-like” structure formed by a central sequence mismatch prevents Ago2-mediated endonucleolytic cleavage and “miR scavenger degradation”, and increases the effectiveness of the blocking action of a miR scavenger.

**Figure 2:**
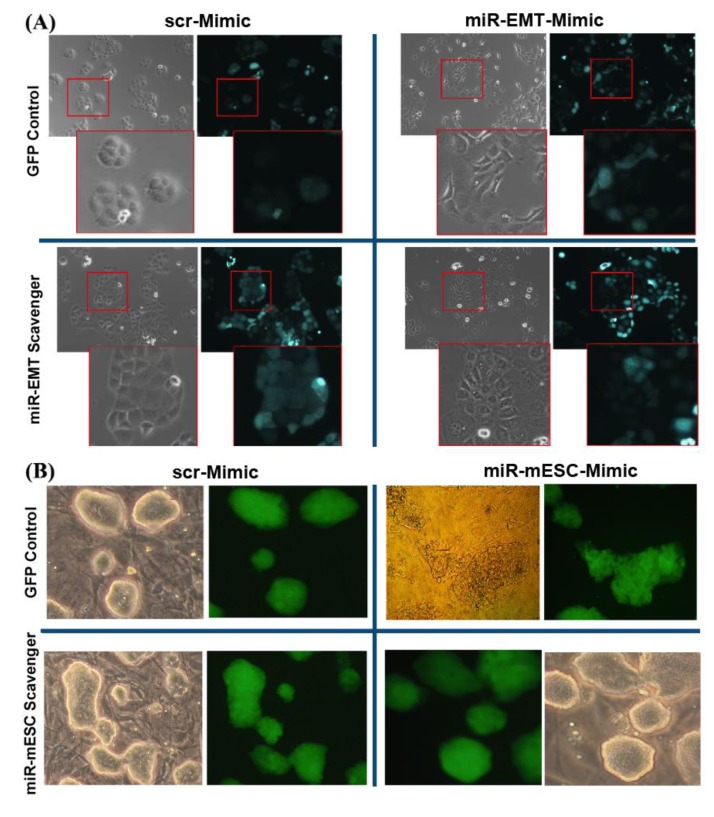
(**A**) “miR-Scavenger” blocks miR induced changes in LA7 and mouse embryonic stem cell (mESC) cell and colony morphologies. “miR-EMT Scavenger” blocks miR-EMT induced changes in LA7 cell and colony morphologies. LA7 cells expressing GFP only, were transfected with miR-scr-Mimic (top left) or miR-EMT Mimic (top right). Dramatic morphological change, loss of an epithelial phenotype and acquisition of a more elongated fibroblast-like morphology (right) were observed after miR-EMT up regulation. The morphological changes induced by miR-EMT up regulation are prevented in LA7 cells expressing “miR-EMT-scavenger” (bottom left) with respect to the control cells or the cells expressing miR scrambled (bottom right). (**B**) “miR-ESC Scavenger” blocks miR-ESC induced changes in mouse embryonic cell and colony morphologies. mESCs expressing GFP only, were transfected with miR-scr-Mimic (top left) or miR-ESC Mimic (top right). Loss of ESC colony morphology (right) was observed after miR-ESC up regulation. The morphological changes induced by miR-ESC up regulation are prevented in ESCs expressing “miR-ESC-scavenger” (bottom right) with respect to the control cells or the cells expressing miR scrambled (bottom left). Phase contrast and copGFP fluorescence images are at 10× and inset at 20× magnification.

**Figure 3:**
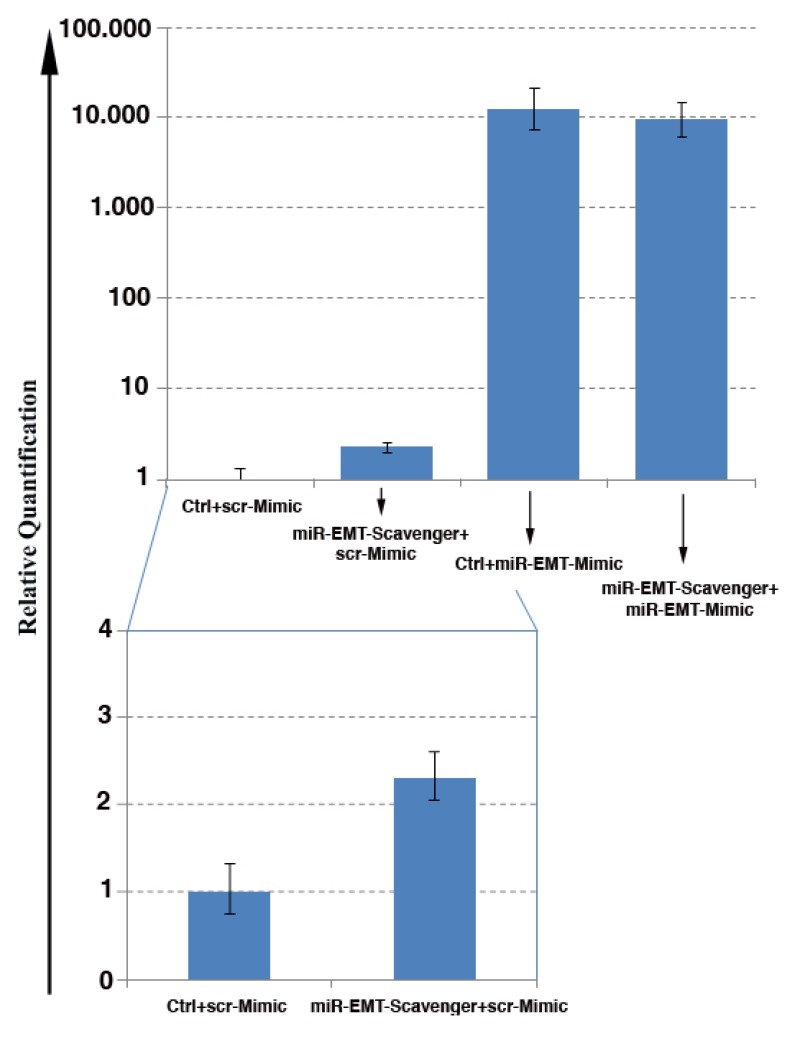
Determination of miR-EMT levels in cells by qPCR analysis. (**A**) Levels of miR-EMT increased approximately 10.000-fold in LA7 cells transfected with 100 nM of miR-EMT Mimic (miR-EMT-Mimic) compared to cells transfected with 100nM scrambled-Mimic (scr-Mimic). The same level of miR-EMT was also detected in LA7 cells transfected with miR-EMT-Mimic oligos and transduced “miR-EMT-scavenger” (miR-EMT-Scavenger + miR-EMT-Mimic) suggesting that the mode of miR-EMT scavenger action is not to eliminate but to sequester miR transcripts in cells. Plot is represented in logarithmic scale using U6 snRNA as an endogenous control. (**B**) Detail of the up-regulation of miR-EMT expression in miR-EMT-scavenger-expressing cells; plot is represented in linear scale using U6 snRNA as an endogenous control. Error bars represent three standard deviations with 95% of confidence. The results are representative of three studies with a *p* < 0.05.

To obtain stable down-regulation or inhibition of microRNA activity, concatamers of 24 “capturing” units (complementary sequences of miRNAs) were designed as “miR scavengers” and cloned into a lentiviral vector according to the method outlined in Figure 1, and described in the Experimental Section (paragraphs from 3.1 to 3.6). As an example, we generated transcripts to block the activity of two unrelated microRNAs we call microRNA-EMT that induces a mesenchymal transition in mammary gland epithelial cells and microRNA-ESC that induces loss of mouse embryonic stem cell (mESC) morphology and pluripotency. We used as an EMT model the LA7 cells, rat mammary gland cancer cells that display stem cell (SC) features [20,21]. LA7 cells exhibit a cobblestone morphology (Figure 2 upper left panel) and express no or a very low levels of miR-EMT. Forced expression of miR-EMT results in a rapid change in LA7 cell morphology, recapitulating an epithelial to a mesenchymal-like transition (Figure 2, upper right panel). To verify the efficiency of miR-EMT scavenger transcripts in blocking the miR-EMT-activity, miR-EMT was simultaneously expressed with miR-EMT-scavenger transcripts in LA7 cells. Cells were transfected with double-stranded RNA oligos (miRIDIAN^TM^ microRNA Mimic, Dharmacon) that mimic miR-EMT activity and transduced with a lentivirus expressing the Green Fluorescent Protein (copGFP) and the “miR-EMT scavenger”. Transduction results in both GFP expression and transcription of the “miR-EMT scavenger”. As controls, LA7 cells were transduced with the lentivirus expressing copGFP only or copGFP and a scrambled (scr) miR-EMT sequence. Our results show that compared with control cells (Figure 2, upper left panel), LA7 cells transduced with “miR-EMT scavenger”, and transfected with miR-EMT Mimic oligos exhibit no change of cell morphology (bottom left panel) suggesting that the “miR-EMT scavenger” effectively inhibits the activity of the endogenous and exogenous miR-EMT. Following miR-EMT up regulation, the level of the expression of miR-EMT was determined to be about 10,000-fold higher (Figure 3, miR-EMT-Mim) than the endogenous level in the control cells (scr-Mim). The same level of miR-EMT was detected in LA7 cells transfected with miR-EMT Mimic oligos and transduced with a lentivirus expressing the “miR-EMT scavenger” (miR-EMT-Mim + “miR-EMT-scav”). Our results suggest that “miR-EMT scavengers” block miR activity by sequestering, rather than eliminating endogenous miR transcripts in cells. A slight up regulation of the miR-EMT transcript level was observed as a consequence of blocking the miR-EMT activity, mostly likely due to an induction of a feed-back regulatory loop that compensates the sequestering effects of scavenger transcripts (Figure 3). This effect is common within regulation networks involving miRNAs in mammalian cells [22,23,24,25,26]. Using a similar approach, we generated transcripts to block the activity of microRNA-ESC that induces loss of pluripotency of mouse embryonic stem cells (mESCs). Mouse ES cells exhibit a 3D morphology of tightly packed cells when grown on mouse embryonic fibroblast cells (Figure 2) and express no or very low levels of miR-mESC (data not shown). Forced expression of miR-mESC results in a rapid change in mESC morphology and colonies of monolayer of cells, and loss of pluriptoency marker Oct4 (data not shown). Mouse ESCs transduced with “miR-ESC scavenger”, and transfected with miR-ESC Mimic oligos exhibit no change of cell and colony morphology (bottom left panel) suggesting that the “miR-ESC scavenger” effectively inhibits the activity of the endogenous and exogenous miR-ESC.

## 3. Experimental Section

The protocol developed to generate “miR-scavengers” is summarized in Figure 1 using miR-21 as an example. The “miR-EMT scavenger” and “miR-mESC scavenger” transcripts were produced according to the following procedure.

### 3.1. Design of the Oligos

Sequence design for blocking miR activity is based on the following modifications of the mature miR sequence. In the mature miR sequence (Figure 1A), the 9th, 10th and 11th nucleotides from the 5' end are substituted with the corresponding complementary nucleotides and the 12th nucleotide is removed (Figure 1C). To obtain the “miR-capturing unit”, the reverse complement of the modified sequence is generated to contain a four nucleotide central mismatch to form a “bulge-like” structure, which prevents the cleavage by Ago2 (Figure 1D). A tandem sequence is generated with two “miR-capturing units” separated by a four-nucleotide spacer (TCAG). The reverse complement (BOT) of the tandem sequence (TOP) is used for making the double-stranded oligos (Figure 1E). The ends are made “sticky” at the 5' of both of the oligos by adding the nucleotides, GCATC to the “TOP” and GATGC to the “BOT” oligos, respectively (Figure 1F).

### 3.2. Concatemerization Step

The oligos designed as described above, are phosphorylated by a polynucleotide kinase (PKN) reaction before concatemerization. The 5' phosphorylated oligos at a concentration of 100 μM are mixed into a annealing reaction containing: 20 µL of TOP oligos, 20 µL of BOT oligos and 10 µL of 5× annealing buffer containing 50 mM Tris pH 7.5–8.0, 250 mM NaCl, 5 mM EDTA. Annealing of the oligos is performed at 95 °C for 4 minutes and room temperature for 15 minutes. The annealed oligos are then used for concatemerization, performed in a total volume of 20 µL with 2 μL of annealed oligos and 1 μL of T4 DNA ligase (400 U/µL) at 37 °C for 15 minutes, and then overnight at 16 °C. The ligation product is loaded on a 2.5% agarose gel. A smear of up to more than 1 Kb indicates a ligation reaction that is efficient (Figure 1G). A portion of the smear around 1 Kb is sliced from the gel and purified using NucleoSpin^®^ Gel and PCR Clean-Up (Machery-Nagel, #740609).

### 3.3. Cloning of the Concatemerized miR Scavenger Sequence

Cloning is performed using a topo-isomerase based PCR-cloning approach. Concatemers are dephosphorylated with alkaline phosphatase and purified using NucleoSpin^®^ Gel and PCR Clean-Up (Machery-Nagel, #740609). The concatemers are eluted into 15 μL and a “fill-in” reaction is performed to fill recessed 3' ends using Taq polymerase (Platinum^®^ Taq DNA Polymerase, Life Technologies, #10966-018) at 72 °C for 10 minutes. For the terminal transferase activity of the Taq Polymerase used for the “fill-in” reaction, a 3' protruding adenosine is added which allows for the cloning with the TOPO^®^ TA Cloning Kit (Life Technologies, #450641).

Colony PCR screening using M13 primers is performed in order to identify positive clones. The presence of concatemerized products and their orientation are confirmed by sequencing. The pCRII shuttle vector is used to clone the “miR-scavenger” sequence into the pCDH-CMV-MCS-EF1-copGFP construct (System Bioscience, # CD511B-1) using the In-Fusion™ Advantage PCR Cloning Kit (Clontech) with modified M13 primers.

### 3.4. Lentivirus Production

Lentiviral particles are produced in HEK-293T cells by transducing the pCDH-miR-Scavenger or empty pCDH vector together with the helper vectors psPAX2 and pMD2.G, in a ratio of 3:2:1 using the Lipofectamine™ 2000 Transfection Reagent (Life Technologies, #11668-019). The supernatant of the culture is harvested after 48 to 72 hours from transduction. Lentiviral particles are concentrated with the PEG-it™ Virus Precipitation Solution (System Biosciences, #LV810A-1) to obtain a high titer concentration.

### 3.5. Transduction of LA7 or mESC Cells

Since the construct used to generate the lentiviral particles express the Green Fluorescent Protein (GFP), lentiviral titration measurements can be performed with fluorescent activated cytometry to validate that most or all of cells express the “miR scavenger” transcript. We determined that cells transduced with a Multiplicity of Infection (MOI) 8 result in an almost 100% transduction efficiency. Transduction is performed essentially by adding the virus preparation directly to the culture medium that was supplemented with hexadimethrine bromide (Sigma, #H9268) at a final concentration of 8 μg/ml. To prevent cellular toxicity, the culture media is replaced after 20 hours.

### 3.6. Inhibition of Mimic Oligo miR Activity by the miR Scavengers

Up-regulation of miR was obtained by transfecting miRIDIAN™ microRNA MIMIC oligos (Dharmacon) into cells and then transducing the cells with a lentivirus expressing “miR scavenger” or GFP alone. Transfection was performed with the INTERFERin^®^ transfection reagent (Polyplus Transfection).

## 4. Conclusions

We developed a method for inhibiting microRNA activity based on stable expression of miR-blocking transcripts, that we call “miR scavengers”. As examples, we demonstrated that stable expression of “miR-EMT or miR-mESC scavengers” inhibited the endogenous and exogenous activity of two different miRs. The cells lines used in this study are models of mammary gland stem cells and pluripotent embryonic stem cells. Our strategy to use “miR scavengers” that contain at least 24 “miR-capturing units” cloned into a lentiviral vector allows for robust quenching of miRNA activity, even in cells expressing high levels of miRs. We simulated this condition in our cell models by inducing high miRNA levels with synthetic oligos and proved that in both cases our “miR scavengers” block the endogenous and exogenous miR activity. Since a larger number of miR-blocking modules (20×) are contained in a single transcript, our method, compared to approaches that have few miRNA “capturing units” (from 6 to 10) is ideal for inhibiting the activity of highly expressed miRNAs in cells that therefore require a higher dosage of miR-scavenger [28]. We obtain this high efficiency not with larger numbers of viral integrations into the genome of cells but with transcripts that contain a large number of miRNA capturing units. The utilization of transcripts with a larger number of “capturing units” reduces the possibility of undesired side effects normally observed with multiple viral integrations.

Since miRs are involved in normal physiological processes and their aberrant expression induces pathological effects in cells, the interest in their use in clinical applications is significant [29,30,31,32]. The intrinsic structure of these small molecules makes it possible to mimic or inhibit their activity for therapeutic strategies and to target a large spectrum of genes related by function or specific pathways. In particular miRs represent a promising field in cancer research and therapy and in regenerative medicine [33,34,35,36]. Lenti-viral expressing miR-scavengers offer the opportunity for investigations into long-term studies *in vivo* and represent a powerful tool to evaluate the role of a microRNA in mouse xenografts [20,21,37].

Bone marrow reconstitution represents another *in vivo* approach in which stable miR down-regulation leads to important insight into microRNA functions. In addition, hematopoietic stem cells stably down-regulating miR-223 phenocopy miR-223 knockout mouse [14]. MiR-145 and miR-146a inhibition recapitulates 5q-myelodysplastic syndrome features [38] while miR-326 down regulation results in auto-immunity phenotype associated with multiple sclerosis [39]. Moreover, the exact contribution of each single miR in the hematopoietic system development was demonstrated using miR-blocking transcripts, with different fluorescence reporters, stably expressed in hematopoietic stem cells used for bone marrow transplantation [18].

The next frontier for the application of this molecular tool, as demonstrated in *Drosophila* [40] is the generation of transgenic organism with spatio-temporal restricted expression of the miR-blocking transcript. In mouse model the “miR-scavenger” approach can be a valid alternative to miR genetic knockout. The ablation of miR genes, in fact, results in some cases in phenotypes linked to different miR functions in different tissues, while a tissue specific block of a specific miR activity, should unveil more precisely its function. First results in this direction demonstrate the effectiveness of this approach in comparison with the genetic knockout [41,42].
